# Phytopreventive antihypercholesterolmic and antilipidemic perspectives of zedoary (Curcuma Zedoaria Roscoe.) herbal tea

**DOI:** 10.1186/s12944-016-0210-y

**Published:** 2016-02-27

**Authors:** Sara Tariq, Muhammad Imran, Zarina Mushtaq, Nosheen Asghar

**Affiliations:** Department of Food Science, Nutrition and Home Economics, Government College University, Faisalabad, Pakistan; Institute of Home and Food Sciences, Faculty of Science and Technology, Government College University, Faisalabad, Pakistan

**Keywords:** *Curcuma zedoaria*, water extract, herbal tea, TPC, DPPH, hypercholesterolemia, hyperlipidemia

## Abstract

**Background:**

Metabolic syndrome is an increasingly prevalent problem, not only in industrialized developed countries, but in developing countries as well. The modern healthcare to reduce the dysfunction of metabolic syndrome is burdened with great problems of unsafe medicines and certain degree of side effects. Medicinal plants and derived component products are becoming increasingly popular in modern society as natural alternatives to synthetic multiple drugs for the treatment of hypercholesterolemia and hypertriglyceridemia. The present research work was carried out to evaluate the zedoary *(Curcuma zedoaria* Roscoe.) herbal tea (ZHT) for antihypercholestrolemic and antilipidemic perspectives in discerning consumers.

**Methods:**

Zedoary rhizome dried powder (ZRDP) after proximate composition analysis was used to prepared ZHT samples as T_1_ (500 mg ZRDP), T_2_ (1 g ZRDP) and T_3_ (1.5 g ZRDP) in 200 mL boiling water for 5 minutes, respectively. ZHT samples were characterized for total phenolic compounds (TPC), DPPH inhibition, total flavonoids, color tonality (L*, a* and b* value), pH, acidity, total soluble solids (TSS) and sensory acceptance. Thirty mild–hypercholestrolemic male human volunteers were randomly allocated to three groups (G_1_, G_2_ and G_3_) and each group consisting of 1o mild–hypercholestrolemic male human subjects. The volunteers were assigned ZHT samples for consecutive two months. The blood drawn for day 0, day 30 and day 60 after an overnight 12 h fast was analyzed for serum parameters such as total cholesterol (TC), high–density lipoprotein cholesterol (HDL–cholesterol), low–density lipoprotein cholesterol (LDL–cholesterol) and triglycerides (TG) concentration.

**Results:**

The ZRDP possessed abundantly the crude protein (13.5 ± 0.68 %), total dietary fiber (21.86 ± 0.71 %), acid detergent fiber (13.22 ± 0.44 %), neutral detergent fiber (18.68 ± 0.53 %) and mineral contents. Highest TPC, DPPH inhibition and total flavonoids values were observed 9.74 ± 0.64 (mg GAE/g DW), 47.28 ± 1.62 (%) and 17.12 ± 0.75 (QE mg/g), respectively in T_3_. L* value was significantly (*p* ≤ 0.05) low for T_3_ samples. In contrast, a* value and b* value was significantly (*p* ≤ 0.05) higher for T_3_ when compared with T_1_ and T_2_. T_3_ samples showed lower pH (5.13 ± 0.13) and higher acidity (0.25 ± 0.08) values than T_1_ (5.64 ± 0.25, 0.17 ± 0.05) and T_2_ (5.42 ± 0.21, 0.21 ± 0.06), respectively. Similarly, an increasing trend in TSS contents was observed. Sensory scores assigned to color, flavor, aroma and overall acceptability attributes varied in a quite narrow range for all ZHT samples. The lowest evaluation scores were recorded for T_3_ samples. The G_3_ showed the more reduction in body weight and BMI during efficacy study as compared to G_1_ and G_2_. The decrease in serum TC for G_1_, G_2_ and G_3_ on day 60 was observed 9 %, 14 % and 17 %, respectively when compared with reference value at day 0. The consumption of T_3_ resulted in significant increase (6.8 %) of HDL-cholesterol after two months. A trend in decrease of serum LDL–cholesterol (5.6 %) and TG (12.5 %) was also observed after consumption of T_3_ at day 60.

**Conclusions:**

The results of the present study conclude that the strong phenolic contents and radical scavenging activity of zedoary rhizome have protective role against hypercholesterolemic and lipidemic conditions.

## Background

The appropriate treatment of different components of metabolic syndrome such as hypercholesterolemia and hypertriglyceridemia require the prescription of synthetic multiple drugs to prevent or to lessen the risk of cardiovascular morbidity and mortality. However, the present drugs available for the treatment of metabolic syndrome are few in number*,* limited in efficacy and have certain degree of side effects. The results of many investigations clearly showed that the inappropriate medication can accelerate the dysfunction of metabolic syndrome and weight gain in susceptible people. In such situation, there should be a clear preference for natural functional foods as alternative medicines which not only lower blood cholesterol but also reduce plasma triglycerides in metabolic syndrome suspected subjects. Plants are considered as a rich source of phytochemicals and many current chemotherapeutic drugs still relies greatly on crude plants, their products and herbal extracts to cure human ailments in developing countries [1]. Family *Zingiberaceae* consisting of about 1400 species and 47 genera has been used in traditional medicine for centuries [2]. *Curcuma zedoaria* also known as white turmeric, kachur and zedoary is a continuing herb belongs to family *Zingiberaceae* which is cultivated all over Asia. The zedoary plant with about 1.2 m height has vertical aerial stems (pseudostems) and straight underground stems known as rhizomes. The zedoary rhizomes color ranges from pale yellow to bright yellow and become brown on age maturity. Dried rhizomes have musky odor with slight camphor smell and a bitter pungent aftertaste. Traditionally, zedoary is being used as antiinflammatory, carminative, antitumor, gastrointestinal stimulant, antiulcer, stomachic, antiallergic, diuretic, hepatoprotective, antinociceptive, demulcent, expectorant, rubefacient and antimicrobial [3–7].

It is well known that the medicinal properties of zedoary depend upon the presence of active chemical components such as terpenoids, flavonoids, phenylpropanoids and sesquiterpenes. Zedoary rhizomes consist of number of bioactive compounds namely zederone, curzerenone, 1,3–hydroxygermacrone, epicurzerenone, curcumol, zedoarol, curcolone, ar–turmerone, zedoarondiol, isocurcumenol, furanodiene, curdione, curcumenol, curcumanolide A, zingiberene, procurcumenol, curzeone, curcumin, curcumenone, curcumanolide B, dehydrocurdione, curzerene, β–turmerone and curcumadiol. The rhizome volatile oil possessed major components as curzerenone, germacrone, camphor and curcumenol [5, 8, 9]. Most of the studies investigating the effects of zedoary and its components on health involve animal models. There are still very few studies done to determine whether the positive results seen in animal studies extend to humans. However, dried zedoary powder has been widely accepted as spices in many conventional recipes. Moreover, zedoary extract has been supplemented in different food products due to presence of potential antioxidant properties [10]. Since plants of family *Zingiberaceae* are well thought-out safe for human use, therefore, these rhizomes can be outstanding candidates for development of new chemotherapeutics and nutraceutical actions. Therefore, keeping in view the medicinal potential and bioactive compounds present in zedoary, the present project was designed to use the zedoary rhizome dry powder (ZRDP) in combating the mild–hypercholesterolemia condition in volunteer human subjects. The main mandate of this study was characterization of ZRDP and its herbal tea (ZHT) for chemical constituents, sensory acceptability, antihypercholestrolemic and antilipidemic perspectives.

## Methods

### Preparation of raw material

Zedoary *(Curcuma zedoaria* Roscoe.) rhizomes, purchased from local super market, were washed to remove the dirt, dust and foreign materials adhered to samples surface. Then, the raw material was dried by using air forced draft oven (Model: DO–1–30/02, PCSIR, Pakistan). The dried material was grounded to fine powder by using a small laboratory grinder (Panasonic, Japan, Model MJ–W176P) and passed through a sieve for further refining. After preparation of powder, it was packed in air–tight plastic jars and stored at 5 ± 1 °C until further analysis.

### Chemical characterization of raw material

Moisture content of zedoary rhizome samples was analyzed by using air forced draft oven. The samples were dried at 105 ± 5 °C to constant weight and calculations were made (Method No. 44–15A) [11]. For determination of crude protein in samples, nitrogen percentage was estimated through Kjeltech Apparatus (Model: D–40599, Behr Labor Technik, GmbH–Germany). The protein was calculated by multiplying percent nitrogen with conversion factor (Method No. 990.03) [12]. Oven dried samples were estimated for crude fat by using Soxtec System (Model: H–2 1045 Extraction Unit, Hoganas, Sweden). 5 g of sample was taken for extraction of crude fat with petroleum ether. After extraction, left over residue was dried until constant weight (Method No. 30–10) [11]. After extraction of fat, samples were studied for crude fiber through Labconco Fibertech (Labconco Corporation Kansas, USA). The digestion of 2 g fat free samples was carried out with 1.25 % H_2_SO_4_ and 1.25 % NaOH. The residue was dried at 130 °C for 2 hours and weighed followed by ignition at 550 ± 15 °C and then cooled for further calculations (Method No. 978.10) [12]. Samples were taken in preweighed crucible for ash content determination and charred on burner till no fumes before incineration in the Muffle Furnace (MF–1/02, PCSIR, Lahore, Pakistan) to obtain white grayish color of residue (Method No. 08–01) [11]. NFE was calculated according to expression: NFE (%) = 100 – (Moisture % + Crude protein % + Crude fat % + Crude fiber % + Total ash %). Total dietary fiber (Method No. 985.29) [12], acid detergent fiber (Method No. 973.18) [12] and neutral detergent fiber [13] contents were analyzed by employing the Megazyme Assay Kit (Megazyme International, Ireland Ltd; Wicklow, Ireland). Concentration of mineral contents was determined by running the diluted ZRDP samples through Atomic Absorption Spectrophotometer (Model: Varian AA–240, Victoria, Australia).

### Development and characterization of ZHT

Preliminary some trials were conducted for the development of ZHT product in laboratory. The purpose was to obtain an acceptable quality product for consumers. After fixing the recipe, different treatments of ZHT were made and evaluated for various quality characteristics. Functional ZHT samples evaluated in the experiments were prepared from boiling of ZRDP as T_1_ (500 mg ZRDP), T_2_ (1 g ZRDP) and T_3_ (1.5 g ZRDP) in 200 mL boiling water for 5 minutes, and strain, respectively. Estimation of total phenolic contents (TPC) was carried out using Folin–Ciocalteu method as described by Singleton et al. [14]. The ability of ZHT samples to scavenge the stable free radical DPPH and convert it into Diphenyl picryl hydrazine was determined by the method described by Mensor et al. [15]. Total flavonoids were estimated using the method of Ordon-ez et al. [16]. Color tonality of product samples was observed by the method of Rocha et al. [17]. For color hue, L* (lightness), a* (–a greenness, +a redness) and b* (–b blueness, +b yellowness) values were recorded using CIE–Lab Color Meter (CIELAB SPACE, Color Tec–PCM, USA). The pH of ZHT samples was measured through electronic digital pH meter (Inolab WTW Series 720). Acidity in ZHT samples was determined by the Method No. 947.05 given in AOAC [12]. The total soluble solids (TSS) in the sample were determined with the help of an Abbe type Refractometer and the values were expressed as degree Brix (°B). A temperature correction was also applied when the temperature was above or below 25 °C.

### Sensory evaluation of ZHT product

The sensory evaluation of ZHT samples was carried out in an adequate room (25 °C) according to the instructions given by Meilgaard et al. [18]. Fourteen judges panel consisting of experienced and untrained panelists was selected for assessing the samples. Each judge gave written informed consent after explanation of risks and benefits of participation prior to the study. Each panelist was offered samples randomly from experimental treatments placed in closed plastic cups labeled with three secret digit codes. Prior to evaluation, the panelists were provided informative instructions and brief definitions of attributes such as color, flavor, aroma and overall acceptability. Each panelist was asked to list their preference on a 9–point Hedonic scale (where 1 = dislike extremely and 9 = like extremely).

### Human subjects, ZHT treatments and serum lipid profile assay

Eligibility in the human study program required willingness and ability to adhere with research program protocol. The study program was designed after the review of ethical guidelines set by parent institute and earlier research studies conducted for evaluation of herbal tea. Thirty mild–hypercholestrolemic male human volunteers ranging in age from 25–40 years old having no diagnosed heart disease were provided a hard copy of informed consent form after expressed an interest as potential participants in the program. They were randomly allocated to three groups (G_1_, G_2_ and G_3_). Each group was consisting of 1o mild–hypercholestrolemic male human subjects. The participants were asked to complete surveys which include questions about dietary intake, physical activities, stress management and any life threatening illness. At the end of survey, the participants were asked to make an appointment for physical assessment on baseline study period. The complete data of participant regarding contact information and method of recruitment was recorded. The participants were given free hand for refuse or answer any questions that were part of the survey. All the information that participant provide, either on paper or in person, was kept confidential. The volunteers were assigned tea bag samples containing 500 mg, 1 g and 1.5 g ZRDP for consecutive two months consumption. The volunteers were advised to prepare the ZHT according to the set procedure and orally consume the ZHT in standard cup twice daily between the meals. The volunteers were further advised to avoid consumption of green or black tea and continue to take routine self-selected diet and perform exercise. The blood drawn for day 0, day 30 and day 60 after an overnight 12 h fast was analyzed for serum parameters. The total cholesterol (TC) was determined by liquid cholesterol CHOD–POP method [19]. The high–density lipoprotein cholesterol (HDL–cholesterol) concentration was analyzed by using HDL–cholesterol kits [20]. Low–density lipoprotein cholesterol (LDL–cholesterol) concentration was assay by following the mathematical expression described by McNamara et al. [21]. The triglycerides (TG) concentration was determined by liquid triglycerides GPO–PAP method [22]. Aspartate aminotransferase (AST) and Alanine aminotransferase (ALT) were determined to assess liver function during the entire study. AST and ALT were measured by dinitrophenylehydrazene (DNPH) method following the procedure of Basuny [23].

### Statistical analysis

The data for chemical characterization of ZRDP and ZHT samples was subjected to statistical analysis to determine the level of significance by using the software package (Minitab® Ver. 8.2.0). The average of the three replicates was reported as the measured value with standard deviation. The Duncan’s multiple range (DMR) test was used to estimate the level of significance that existed between the mean values. The effect analysis of ZHT on serum lipid profile of mild–hypercholestrolemic human subjects was carried out in triplicate and calculated the significant differences among means at a probability level of 5 % [24].

## Results and discussion

### Physico-chemical composition and sensory acceptability of ZRDP and ZHT

The mean values for the moisture, crude protein, crude fat, crude fiber, ash and NFE contents of ZRDP were found 10.74 ± 0.52 (g/100 g DM), 13.5 ± 0.68 (g/100 g DM), 3.64 ± 0.24 (g/100 g DM), 2.32 ± 0.18 (g/100 g DM), 6.21 ± 0.41 (g/100 g DM) and 63.58 ± 0.66 (g/100 g DM), respectively. ZRDP possessed total dietary fiber (21.86 ± 0.71 g/100 g DW), acid detergent fiber (13.22 ± 0.44 g/100 g DW) and neutral detergent fiber (18.68 ± 0.53 g/100 g DW) contents, respectively. The minerals calcium, copper, zinc, magnesium, potassium, phosphorous, sodium and iron were ranged 1.92 ± 0.12 g/kg DM, 6.38 ± 0.14 mg/kg DM, 104.5 ± 1.38 mg/kg DM, 2.42 ± 0.11 g/kg DM, 22.65 ± 0.26 g/kg DM, 3.10 ± 0.13 g/kg DM, 0.28 ± 0.09 g/kg DM and 312.45 ± 2.88 mg/kg DM, respectively in ZRDP samples. There is very scant information available regarding the chemical composition of the true zedoary rhizome in the literature during the past decade [25]. Zedoary (bulb) and zedoary (finger) possessed the moisture contents 7.30 ± 0.45 % and 6.28 ± 0.08 %, respectively [26]. In another study, moisture content in zedoary rhizome samples was noted 5.35 ± 0.94 % [27]. Proximate composition showed that the rhizomes of zedoary contained 14.85 % protein on moisture free basis while organic and inorganic phosphorus and calcium contents of rhizomes were found very high in concentration [28]. Total ash content in rhizome was found 7.5 ± 0.16 % [27] while other study concluded that the zedoary rhizome yields not more than 6.64 % percent of ash [29]. The changes in proximate composition in comparison to earlier reported values may be due to climate conditions, ripening stages, soil type, soil condition and irrigation regime [30, 31]. Other differences in concentration could be explained by adulteration after harvest, processing or transport [32].

**Fig. 1:**
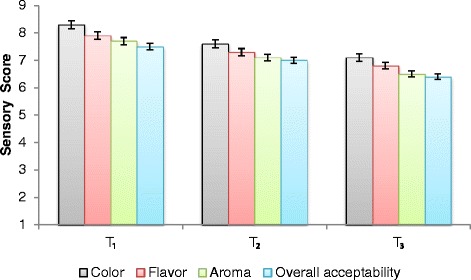
Organoleptic evaluation of zedoary herbal tea for sensory attributes (color, flavor, aroma and overall acceptability)

The significant results of ZHT samples analyzed for physico-chemical analysis (total phenolic contents, DPPH inhibition, total flavonoids, color tonality, pH, acidity and total soluble sugars) have been presented in Table 1. Results indicate that the total phenolic, DPPH inhibition and total flavonoids were gradually increased on the incorporation of ZRDP from 0.5 mg to 1.5 g in ZHT boiling water. Maximum concentration for these parameters was observed 9.74 ± 0.64 (mg GAE/g DW), 47.28 ± 1.62 (%) and 17.12 ± 0.75 (QE mg/g), respectively in T_3_. The incorporation of ZRDP in ZHT influenced the color tonality in the term of L* value, a* value and b* value. L* value was significantly (*p* ≤ 0.05) decreased for T_3_ samples. In contrast, a* value and b* value was significantly (*p* ≤ 0.05) increased for T_3_ samples when compared with T_1_ and T_2_. T_3_ samples showed lower pH (5.13 ± 0.13) and higher acidity (0.25 ± 0.08) values than T_1_ (5.64 ± 0.25, 0.17 ± 0.05) and T_2_ (5.42 ± 0.21, 0.21 ± 0.06). Similarly, an increasing trend in TSS contents was observed with an increase in supplementation of ZRDP in ZHT boiling water. The results of organoleptic evaluation point out the differences between ZHT samples acceptability. Figure 1 indicated that the gradual increase in ZRDP concentration in boiling water affected the sensory characteristics of ZHT samples. The sensory attributes color and flavor obtained higher scores as compared to aroma. Overall, average sensory scores assigned to color, flavor, aroma and overall acceptability attributes varied in a quite narrow range for all ZHT samples. The lowest evaluation scores were recorded for T_3_ samples.

**Table 1:** Physico-chemical composition of zedoary herbal tea

Treatments	Physico-chemical components								
	TPC (mg GAE/g DW)	DPPH Inhibition (%)	TFC (QE mg/g)	Color tonality			pH	acidity	TSS
				L* value	a* value	b* value			
T_1_	5.90 ± 0.42^c^	36.57 ± 1.24^c^	10.76 ± 0.58^c^	22.36 ± 1.52^a^	7.78 ± 0.51^c^	5.75 ± 0.23^c^	5.64 ± 0.25^a^	0.17 ± 0.05^c^	2.13 ± 0.15^c^
T_2_	7.35 ± 0.56^b^	42.28 ± 1.45^b^	14.39 ± 0.66^b^	21.50 ± 1.36^b^	8.98 ± 0.55^b^	6.99 ± 0.35^b^	5.42 ± 0.21^b^	0.21 ± 0.06^b^	3.86 ± 0.21^b^
T_3_	9.74 ± 0.64^a^	47.28 ± 1.62^a^	17.12 ± 0.75^a^	18.91 ± 1.28^c^	11.35 ± 0.63^a^	8.63 ± 0.42^a^	5.13 ± 0.13^c^	0.25 ± 0.08^a^	4.90 ± 0.30^a^

*Values represent the mean ± standard deviation; n = 3*

^*a,b,c*^ *Means in a column with different superscripts were significantly different (p ≤ 0.05)*

*TPC = Total phenolic contents; DPPH = Diphenyl picryl hydrazine; TFC = Total flavonoids contents; TSS = Total soluble solids*

Various studies support the findings of present research regarding the physico-chemical composition of ZHT samples. TPC in hydroethanolic extract of *Curcuma zedoaria* was found to be 34.45 ± 1.9 expressed as mg/g equivalent of gallic acid [33]. *Curcuma longa* and *Curcuma aromatica* exhibited similar levels of TPC, whereas *Curcuma zedoaria* showed slightly lower concentration [34]. Antioxidant properties have been found in *Curcuma zedoaria*. The aqueous extract of fresh and dry *Curcuma zedoaria* rhizome exhibited DPPH radical scavenging activity in the range of 39–41 % which is very similar to values reported in the present study [35]. *In vitro* antioxidant activity, IC_50_ value for zedoary hydroethanolic extract was found to be 930 ± 16.35 for DPPH method [33]. Similarly, the concentration of 100 microg/mL of water extract of rhizome part of the zedoary plant material exhibited 98.95 % inhibition of DPPH free radicals. DPPH free radical scavenging activity of solvent extracts were high in the order of ethanol, ethylacetate, ether, water, and hexane fractions, *Curcuma longa* showed greater DPPH free radical scavenging activity than those of the *Curcuma aromatic* and *Curcuma zedoaria* [34]. The antioxidant activity of n-hexane, n-butanol and aqueous *Curcuma zedoaria* soluble fractions was found very less as compared to chloroform and ethyl acetate soluble fractions [36]. The presence of bioactive compounds in solvent extract shows potent antioxidant activity in product [37]. The key role of phenolic compounds as scavengers of free radicals is emphasized in several reports [38, 39]. It has been observed that the phenolic compounds are very important plant constituents because of their scavenging ability due to their hydroxyl groups [40]. The hydroethanolic extract of *Curcuma zedoaria* rhizome showed antioxidant activity which demonstrates only the presence of the alkaloids [33]. Many medicinal plants contain large amounts of antioxidants such as polyphenols which have been widely used as additives to avoid the degradation of foods. Also, polyphenols have an important role in preventing a variety of stress-related diseases because these are closely related to the active oxygen and lipid peroxidation [41]. Phenolic antioxidants are products of secondary metabolism in plants, and the antioxidant activity is mainly due to their redox properties and chemical structure, which can play an important role in chelating transitional metals, inhibiting lipoxygenase and scavenging free radicals [42].

The main active constituents of zedoary rhizomes are considered coloring matters (curcumin, demethoxycurcumin and *bis*-demethoxycurcumin) and volatile oil (turmerone, *ar*turmerone and zingiberene) contents [43, 44] which have widely applications in food industries [45]. The color tonality for T_3_ indicates that this was the darkest product compared to the T_1_ and T_2_. It was followed by T_2_ and T_1_ being the lightest ones. The boiling of ZRDP enhances the loss of soluble sugars in the cooking water which seems true in case of T_3_. The presence of more soluble sugars in T_3_ leads towards the occurrence of Maillard reaction which could cause browning and darkening of the tea samples. The study indicates that processing can be optimized in order to obtain ground *Curcuma* rhizome with desirable properties to satisfy different markets, such as a product with higher intensity of red for Asian countries and with higher intensity of yellow for other global markets [46]. The change in the pH and acidity between different ZHT samples might be due to degradation of reducing sugars, formation of acidic components and various by-products of these acids [47]. Sensory evaluation showed that ZHT T_1_ and T_2_ samples were better in appearance, odor and overall acceptability than T_3_ samples. Inspite of the efforts to collect literature in this regard, very limited relevant literature could be collected. The acceptability analysis of *Curcuma longa* powder indicates that the samples with bright yellow color were more appealing to eyes as scored 8 in the hedonic scale and the flavor characteristic obtained score 7 from sensory panelists [48]. There were no significant differences among the turmeric: ginger based drinks with respect to color, taste, aroma and general acceptability [49].

### ZHT consumption, anthropometric measurements and human plasma lipidic profile

Results presented in Table 2 indicated that there was slight difference in body weight and BMI values of mild–hypercholestrolemic human subjects due to consumption of ZHT samples for consecutive two months. The body weight of subjects ranged from 78.40 ± 5.54 kg to 72.76 ± 4.67 kg from day 0 to the end of study. However, the G_3_ showed the more reduction in body weight during efficacy study as compared to G_1_ and G_2_. BMI values ranged from 27.74 ± 2.03 to 25.98 ± 1.46 from day 0 to day 60. Although, all treatments were effective in reducing BMI value but T_3_ showed more pronounced effect. The mean values of BMI noted for G_1_, G_2_ and G_3_ were 26.82 ± 1.72, 26.14 ± 1.34 and 25.98 ± 1.46, respectively at day 60. Fresh zedoary rhizomes were minced and dried and the resulting meal was given to weanling rats at 400 g/kg diet. All the rats lost weight rapidly. This same zedoary meal was given to one-day-old chicks at 100 and 200 g/kg diet. All the chicks survived the test period (20 days), but body weight, food intake and efficiency of food conversion decreased with increase in the level of zedoary meal in the diet [50] which support the results found in the present study regarding the loss in body weight after consumption of ZHT. The data collected from ZHT consumers showed that most of the subjects pointed the improved appetite and digestion while none of volunteers feel hyperacidity and gastric pain after taking the ZHT. The root powder at a dose level of 200 mg/kg reduced the gastric pH, free acid, total acid and ulcer index significantly which provide justification that the zedoary root is effective in affording protection against hyperacidity and gastric ulcers [51].

**Table 2:** Impact of zedoary herbal tea consumption on weight (kg) and body mass index (BMI) of mild–hypercholestrolemic human subjects

Analysis duration	Weight (kg)			Body mass index (BMI)		
	G_1_	G_2_	G_3_	G_1_	G_2_	G_3_
0 day	78.40 ± 5.54^a^	76.56 ± 5.87^a^	75.61 ± 6.20^a^	27.74 ± 2.03^a^	27.06 ± 1.95^a^	26.92 ± 1.54^a^
30 days	77.12 ± 4.86^a^	75.23 ± 5.15^a^	74.30 ± 5.21^a^	27.36 ± 1.97^a^	26.78 ± 1.55^a^	26.24 ± 1.29^a^
60 days	75.95 ± 4.32^b^	73.75 ± 4.46^b^	72.76 ± 4.67^b^	26.82 ± 1.72^a^	26.14 ± 1.34^a^	25.98 ± 1.46^a^

*Values represent the mean ± standard deviation; n = 3*

^*a,b*^ *Means in a column with different superscripts were significantly different (p ≤ 0.05)*

*G*_*1*_ *= Provided T*_*1*_ *Diet; G*_*2*_ *= Provided T*_*2*_ *Diet; G*_*3*_ *= Provided T*_*3*_ *Diet*

**Fig. 2:**
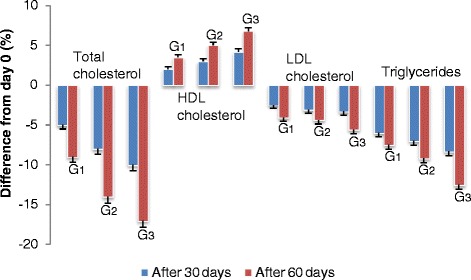
Effect of zedoary herbal tea consumption on percent change in serum lipid profile in mild-hypercholesterolemic human subjects

The effect of experimental ZHT consumption on plasma lipidic profile of mild–hypercholestrolemic human subjects is shown in Table 3. The results regarding percent change in plasma lipidic profile as a result of intake of different experimental ZHT samples have been illustrated in Fig. 2. The results showed that TC, LDL–cholesterol and TG was decreased significantly (p ≤ 0.05) by consuming ZHT samples in experimental subjects at day 60 when compared to the beginning of the study. The decrease in serum TC in human subjects fed on T_1_, T_2_ and T_3_ samples was observed 9 %, 14 % and 17 % on day 60, respectively from the reference value at day 0. There was no significant change in HDL-cholesterol by using T_1_ at day 60. However, the consumption of T_3_ resulted in significant increase (6.8 %) of HDL-cholesterol after two months. A trend in decrease of serum LDL–cholesterol (5.6 %) and TG (12.5 %) was also observed after consumption of T_3_ samples at day 60. The ZHT samples consumption was found to reduce the TC and TG effectively in mild–hypercholesterolemic conditions. It was reported that *Curcuma zedoaria* inhibited 50.60 % platelet activating factor binding to rabbit platelets at a concentration of 200 mg/mL [52]. Means depicted in Table 3 for AST showed non-significant differences (*p* ≥ 0.05) from 34.88 ± 1.23 to 33.95 ± 1.11 U/L and 34.56 ± 0.92 to 33.21 ± 1.16 U/L at initiation and termination of study in G_1_ and G_3_, respectively. Similarly, ALT values were recorded ranging from 37.28 ± 1.41 to 36.45 ± 1.21 U/L and 37.72 ± 1.32 to 35.93 ± 1.06 U/L at 0 and 60 days in respective mild-hypercholesterolemic subject groups, correspondingly. It is worth mentioning that overall means of AST and ALT remained in safe range predicting proper liver functioning and indicating that the ZHT consumption is friendly for mild-hypercholesterolemic subjects. The survey of 32 books published in Brazil between 1998 and 2008, resulting in a list of 85 medicinal plants species belonging to 53 families including *Curcuma zedoaria* (*Zingiberaceae*) indicates the consumption of these plants for the possible treatment of hyperlipidemia, hypercholesterolemia and/or atherosclerosis [53]. Twelve Thai selected plants including *Curcuma zedoaria* supplemented as spices and ingredients in various types of Thai foods possessed multiple sites of action that were possibly responsible for their cholesterol-lowering effect in the in-vivo model [54]. The zedoary extract at a dose of 200–400 mg/kg b/w was found to be effective in reducing TC levels (17.1 %–19.65 %) after 12 days of pre-treatment which indicates antihyperlipidemic activity. However, no significant changes were seen on LDL–cholesterol, VLDL and HDL–cholesterol levels [33]. The supplementation and oral administration of *Curcuma zedoaria* in male Wistar rats diet resulted in low levels of total lipids, TC, TG, phospholipids and thiobarbituric acid reactive substances (TBARS) in the liver which suggests that *Curcuma zedoaria* may have recuperative effects for hypercholesterolemia [55]. Herbal preparations of *Curcuma zedoaria* lowered the levels of serum TC, phospholipids and TG to varying extents in Triton-induced hyperlipidemic rats [56]*.* Administration of dried leaf powder leads towards decrease in levels of serum glucose, TC, TG and LDL–cholesterol levels in Wistar rats [57]. *Curcuma’s* cholesterol-lowering actions include interfering with intestinal cholesterol uptake, increasing the conversion of cholesterol into bile acids and increasing the excretion of bile acids via its choleretic effects [58]. Among the plant extracts, *Curcuma* showed potent antioxidant activity which might be due to the presence of high phenolic and flavonol contents such as curcumin which help to reduce the blood cholesterol, prevents LDL peroxidation, inhibits platelet aggregation and suppress thrombosis [59]. Metabolic risk factors, particularly serum concentration of TGs and HDL–cholesterol have been reported to improve most with weight loss in men [60] which seems true in the present research study as slight change in body weight of ZHT consumers was related with beneficial effect on dyslipidemia. The medicinal plants such like zedoary may be broadly applied in modern phytotherapy once they have been clinically and experimentally tested [61]. The natural extracts of *Curcuma zedoaria* can be explained in the field of pharmaceutical areas for their uses in modern health care as phytoprotectants [62]. The rhizomes of the family *Zingiberaceae* are a vegetable widely used in many Asian countries and their medicinal functions have been broadly discussed and accepted in many traditional recipes [63]. Therefore, this study is a positive demonstration of the utility of zedoary for food and medicinal uses.

**Table 3:** Effect of zedoary herbal tea on serum lipid parameters in mild-hypercholesterolemic human subjects

Serum parameter	Treatment	Analysis period		
		0 day	30 days	60 days
Total cholesterol (mg/dL)	G_1_	218.42 ± 3.16^a^	207.40 ± 2.94^b^	198.60 ± 2.53^c^
	G_2_	222.2 ± 3.08^a^	204.32 ± 2.63^b^	190.92 ± 2.15^c^
	G_3_	225.50 ± 2.94^a^	202.70 ± 2.96^b^	186.50 ± 3.55^c^
HDL-cholesterol (mg/dL)	G_1_	48.26 ± 2.12^a^	49.22 ± 2.25^a^	49.68 ± 2.44^a^
	G_2_	47.5 ± 2.24^a^	48.93 ± 2.35^a^	49.87 ± 2.57^a^
	G_3_	49.78 ± 2.1^c^	51.87 ± 2.75^b^	53.16 ± 2.22^a^
LDL-cholesterol (mg/dL)	G_1_	116.32 ± 3.11^a^	113.41 ± 2.65^b^	111.55 ± 3.15^b^
	G_2_	119.18 ± 2.28^a^	115.60 ± 2.12^b^	113.93 ± 2.97^b^
	G_3_	118.45 ± 2.76^a^	114.54 ± 2.14^b^	111.81 ± 1.95^c^
Triglycerides (mg/dL)	G_1_	160.38 ± 3.44^a^	150.75 ± 3.54^b^	148.35 ± 2.61^b^
	G_2_	164.21 ± 3.17^a^	152.62 ± 2.26^b^	149.10 ± 3.12^b^
	G_3_	168.70 ± 2.92^a^	154.69 ± 2.78^b^	147.61 ± 2.56^c^
AST (U/L)	G_1_	34.88 ± 1.23^a^	34.15 ± 1.14^a^	33.95 ± 1.11^a^
	G_2_	34.72 ± 1.17^a^	33.98 ± 1.10^a^	33.70 ± 1.12^a^
	G_3_	34.56 ± 0.92^a^	33.75 ± 1.08^a^	33.21 ± 1.16^a^
ALT (U/L)	G_1_	37.28 ± 1.41^a^	36.87 ± 1.34^a^	36.45 ± 1.21^a^
	G_2_	37.46 ± 1.27^a^	36.57 ± 1.26^a^	36.10 ± 1.12^a^
	G_3_	37.72 ± 1.32^a^	36.54 ± 1.18^a^	35.93 ± 1.06^a^

*Values represent the mean ± standard deviation; n = 3*

^*a,b,c*^ *Means in a row with different superscripts were significantly different (p ≤ 0.05)*

*HDL High Density Lipoprotein, LDL Low Density Lipoprotein*

*AST Aspartate aminotransferase, ALT Alanine aminotransferase*

*G*_*1*_ *= Provided T*_*1*_ *Diet; G*_*2*_ *= Provided T*_*2*_ *Diet; G*_*3*_ *= Provided T*_*3*_ *Diet*

## Conclusions

The results of the present study conclude that the strong phenolic contents and radical scavenging activity of zedoary rhizome have protective role against dysfunction of metabolic syndrome. The information from the present study can be applied for the industrial production of zedoary supplemented food products. Further studies are needed to investigate the storage stability of zedoary supplemented products and their role as therapeutic agent in preventing or slowing down the progress of ageing and age associated oxidative stress related degenerative diseases in women subjects with and without allied conditions of menstruation, pregnancy or breastfeeding stage. Randomized double blind placebo control studies involving zedoary consumption should be considered for healthy and diabetic subjects in future. It is also proposed that patients on blood-thinning medications or blood pressure medications should also speak with a health care provider before taking zedoary.
